# The effects of C*osmos caudatus* (ulam raja) on dynamic and cellular bone histomorphometry in ovariectomized rats

**DOI:** 10.1186/1756-0500-6-239

**Published:** 2013-06-24

**Authors:** Norazlina Mohamed, Zulaikha Sahhugi, Elvy Suhana Mohd Ramli, Norliza Muhammad

**Affiliations:** 1 Department of Pharmacology, Universiti Kebangsaan Malaysia Medical Center, Jalan Raja Muda Abdul Aziz, 50300, Kuala Lumpur, Malaysia; 2 Department of Anatomy, Universiti Kebangsaan Malaysia Medical Center, Kuala Lumpur, Malaysia; 3 Department of Biomedical Sciences, Faculty of Health Sciences, Kuala Lumpur, Malaysia

**Keywords:** *Cosmos caudatus*, Osteoporosis, Femur, Bone histomorphometry, Ovariectomized rats

## Abstract

**Background:**

*Cosmos caudatus* is a local plant which has antioxidant properties and contains high calcium. It is also reported to be able to strengthen the bone. This report is an extension to previously published article in Evidence Based Complementary and Alternative Medicine (doi:10.1155/2012/817814). In this study, we determined the effectiveness of *C. caudatus* as an alternative treatment for osteoporosis due to post-menopause by looking at the dynamic and cellular paramaters of bone histomorphometry.

**Methods:**

Forty female Wistar rats were divided into four groups i.e. sham operated, ovariectomized, ovariectomized treated with calcium 1% *ad libitum* and ovariectomized force-fed with 500 mg/kg *C. caudatus* extract. Treatment was given six days a week for eight weeks.

**Results:**

Dynamic and cellular histomorphometry parameters were measured. *C. caudatus* increased double-labeled surface (dLS/BS), mineral appositional rate (MAR), osteoid volume (OV/BV) and osteoblast surface (Ob.S/BS). *C. caudatus* also gave better results compared to calcium 1% in the osteoid volume (OV/BV) parameter.

**Conclusions:**

*C. caudatus* at the 500 mg/kg dose may be an alternative treatment in restoring bone damage that may occur in post-menopausal women.

## Background

This paper reported the findings which were an extension to an article recently published [1].

Low bone mass and deterioration of bone tissue microarchitectural which are observed in osteoporosis may eventually lead to increase bone fragility and tendency to fracture [2,3]. Osteoporosis has many negative impacts on public health by increasing death rates and economic costs due to the impact of the fracture [2]. Increasing age is a major risk for osteoporosis and fractures [4].

To date, there are several treatments available to treat osteoporosis such as bisphosphonates, calcitonin, estrogen and/or hormone therapy, selective estrogen receptor modulator, anabolic agents and others [5]. However, there are many studies reporting that these treatments have side effects such as estrogen treatment can pose risk of breast cancer [6], ovarian and endometrial cancer [7] to the osteoporosis patients even though it can reduce the fracture of hip, vertebrae and other bones [8].

Natural products have been proven to affect bone metabolism by involving a complex balance between deposition, mineralization and resorption of bone matrix [9]. Natural substances that were often studied and were observed to have osteoprotective effects were phytoestrogens such as ferutinin [10], genistein [11] and 8-prenylnaringenin [12].

*Cosmos caudatus* or better known among locals as ulam raja is a popular herb in Malaysia [13]. Methanol extract of *C. caudatus* was found to have antioxidant activity [14,15]. In addition, *C. caudatus* was also found to have antifungal activity [16] and can stimulate bone formation [17]. In analogy with other vegetal substances already demonstrated to mime osteoprotective functions as described above, *C. caudatus* was considered to be able to act on bone tissue. Thus, this study was done to determine the effects of *C. caudatus* on dynamic and cellular bone histomorphometry of ovariectomized rats which will serve as a model for post-menopausal women.

## Methods

### Animals and treatment

This study utilized thirty-two young adult (3 months) female Wistar rats, which were obtained from the Laboratory Animal Resource Unit, Faculty of Medicine, Universiti Kebangsaan Malaysia. The rats, weighing 190 g-260 g, were randomly assigned to four groups with eight rats in each group. Group 1 was sham operated (Sham) while the second group was ovariectomized control group (OVX). The third and fourth groups were ovariectomized and treated with calcium 1% (Ca) *ad libitum* and force-fed with 500 mg/kg *C. caudatus* extract (CC) respectively. The ovariectomy and sham operation protocol was carried out as previously described [18]. Rats were left recuperating for 1 week before commencing the treatment. Treatment was given six days a week for eight weeks. All rats were injected with two doses of calcein nine days and two days before sacrificed. The study was approved by the Universiti Kebangsaan Malaysia Animal Ethics Committee with the approval code of PP/FAR/2008/NORAZLINA/12-AUGUST/225-SEPT-2008-AUG-2009.

### Diet, *Cosmos caudatus* and calcium 1%

All rats received normal rat chow obtained from Gold Coin, Malaysia. Water extraction method was used to obtain 500 g/300 ml concentration of *C. caudatus* aqueous extract. Extraction was carried out by School of Chemical Sciences & Food Technology, Faculty of Science and Technology, Universiti Kebangsaan Malaysia as previously described [19]. The 500 mg/kg dose was prepared by mixing *C. caudatus* with deionized water in ratio 3:7. Calcium 1% solution was prepared by mixing 1 g of hemicalcium lactic acid (Sigma Chemical CO., USA) with 100 ml deionized water.

### Bone histomorphometry

After 8 weeks of treatment, rats were sacrificed via euthanasia with diethyl ether. The left femora were removed and the distal parts were fixed in 70% ethanol. Cellular parameters measured were osteoid volume (OV/BS, osteoid volume over bone surface), osteoid surface (OS/BS, osteoid surface over bone surface), osteoclast surface (Oc.S/BS, osteoclast surface over bone surface), osteoblast surface (Ob.S/BS, osteoblast surface over bone surface) and eroded surface (ES/BS, eroded surface over bone surface). Bone samples were decalcified according to the method described by Hermizi et al. [20]. In addition, undecalcified bone samples were used to measure dynamic parameters i.e. single labeled surface (sLS/BS, single labeled surface over bone surface), double-labeled surface (dLS/BS, double-labeled surface over bone surface), mineralized surface (MS/BS, mineralized surface over bone surface), mineral appositional rate (MAR), and bone formation rate (BFR/BS, bone formation rate over bone surface). Preparation of bone samples and measurement techniques were carried out as previously described [20].

### Statistical analysis

All data were subjected to normality test using the Kolmogorov test. For normally distributed data, the statistical test used was analysis of variance, followed by Tukey’s honestly significance difference test. While non-normally distributed data were analysed using Mann–Whitney and Kruskal Wallis tests. Data analysis was performed using Statistical Package for Social Sciences (18.0.1; SPSS, Inc., Chicago, IL) software. Results were expressed as mean ± standard error of the mean (SEM).

## Results

**Figure 1:**
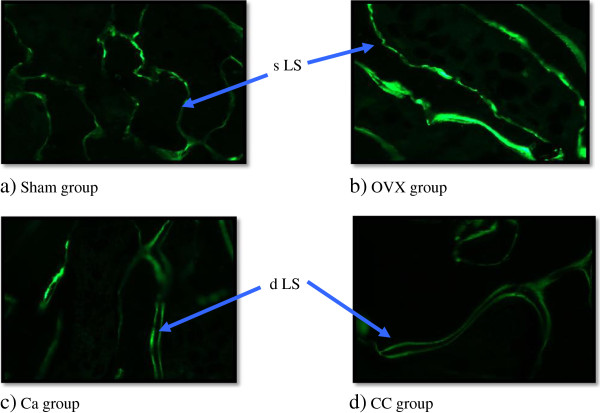
**Photomicrographs of trabecular bone labelled with calcein.** Arrows indicating dLS (double-labeled surface) and sLS (single-labeled surface).

Figure 1 shows the photomictorographs of the trabecular bone of the distal part of femurs analysed using fluorescence microscope. Calcein acts as a fluorescent marker which labels the bone surface thus the double-labeled surface (dLS) and single-labeled surface (sLS) parameters may be determined. From the photos, it was observed that the OVX group had more sLS than dLS while the Ca and CC groups had more dLS than sLS.

Table 1 shows the dynamic bone histomorphometric results. The Ca and CC groups showed significant increase (p < 0.05) in dLS/BS compared to the sham group. For mineral appositional rate parameter (MAR), the Ca and CC groups showed a significant increase (p < 0.05) compared to the sham and OVX group.

**Table 1:** **The effects of *Cosmos caudatus *and calcium on dynamic histomorphometry parameters in ovariectomized rats**

**Dynamic parameters**	**Sham**	**OVX**	**Ca**	**CC**
Single labeled surface (%)	75.44 ± 4.00	78.99 ± 3.35	73.8 ± 6.33	73.1 ±6 .33
Double-labeled surface (%)	18.5 ± 3.33	24.68 ± 5.08	28.38 ± 2.78^*^	38.9 ± 4.10^*^
Mineralized surface (%)	58.69 ± 1.10	63.78 ± 1.18	60.55 ± 2.86	63.55 ± 3.10
Mineral appositional rate (μm/day)	1.52 ± 0.12	1.52 ± 0.13	3.20 ± 0.38^#*^	3.60 ± 0.09^#*^
Bone formation rate (μm^3^/μm^2^/day)	1.00 ± 0.09	1.08 ± 0.02	1.58 ± 0.27	1.48 ± 0.29

*Sham* sham-operated rats.

*OVX* ovariectomized rats.

*Ca* ovariectomized rats supplemented with 1% calcium.

*CC* ovariectomized rats supplemented with 500 mg/kg *Cosmos caudatus.*

* significant vs sham group (p < 0.05).

# significant vs OVX group (p < 0.05).

Data is presented as mean ± S.E.M.

The results for the cellular parameters are shown in Table 2. The osteoblast surface (Ob.S/BS) of the Ca and CC groups were increased significantly (p < 0.05) compared to the OVX group. Osteoid volume (OV/BS) was significantly increased (p < 0.05) in the sham and CC groups compared to the OVX group. In addition, osteoid volume of CC group was also higher (p < 0.05) compared to the Ca group. No significant differences were seen in the other parameters.

**Table 2:** **The effects of *Cosmos caudatus *and calcium on cellular histomorphometry parameters in ovariectomized rats**

**Cellular parameters**	**Sham**	**OVX**	**Ca**	**CC**
Osteoblast surface (%)	30.92 ± 8.78	8.75 ± 5.15*	55.32 ± 10.2^#^	70.4 ± 7.78^#^
Osteoclast surface (%)	52.43 ± 12.59	65.56 ± 13.04	62.96 ± 6.68	36.12 ± 1.11
Eroded surface (%)	12.32 ± 6.00	38.89 ± 14.70	36.53 ± 9.15	22.62 ± 4.66
Osteoid volume (%)	13.13 ± 1.92	4.54 ± 2.46*	8.66 ± 1.07	18.27 ± 0.53^#@^
Osteoid surface (%)	72.95 ± 2.66	51.29 ± 3.43	63.08 ± 5.62	71.85 ± 7.09

*Sham* sham-operated rats.

*OVX* ovariectomized rats.

*Ca* ovariectomized rats supplemented with 1% calcium.

*CC* ovariectomized rats supplemented with 500 mg/kg *Cosmos caudatus.*

* significant vs sham group (p < 0.05).

# significant vs OVX group (p < 0.05).

@ significant vs Ca 1% (p < 0.05).

Data is presented as mean ± S.E.M.

## Discussion

Ovariectomy caused a state of estrogen deficiency which may lead to bone loss. The use of rats as a model for postmenopausal osteoporosis has been discussed and significant bone loss may be seen as early as 14 days in the proximal tibial metaphysis [21]. The ovariectomy-induced osteopenia observed in rats are similar to skeletal responses in post-menopausal women [22].

In this study, the ovariectomized group did not differ to sham-operated group in terms of dynamic histomorphometry parameters (Table 1). These findings were in agreement with our previous study except in one parameter. The previous study showed that the ovariectomized rats had lower mineral appositional rate compared to the sham-operated group [23]. In terms of cellular histomorphometry parameters, ovariectomy caused reduction in osteoid volume as compared to the sham group. Other parameters were not significantly different compared to sham even though declining values were observed. However, in another study it was observed that cellular parameters deteriorated in the ovariectomized group as compared to the sham group [24]. The discrepancies seen in the histomorphometry parameters between ovariectomized and sham-operated groups were surprising and it may be attributed to the small sample size used in our study. In addition, the technique used in measuring the histomorphometric parameters may not be sensitive since others have found that the use of high-throughput technique such as microcomputed tomography was able to yield more significant results [25].

The effects of *C. caudatus* on bone biochemical markers were previously investigated in which we used the doses of 100, 200 and 300 mg/kg. It was observed that all doses of *C. caudatus* were able to prevent the increase in interleukin-1 and pyridinoline in the ovariectomized group. However, no significant changes were seen in the bone histomorphometry parameters (unpublished data).

Following that, we proceeded with a higher dose i.e. 500 mg/kg and look at the bone histomorphometry paramaters. Parts of the results from this study were previously published in which we observed *C. caudatus* was able to improve some of the structural bone histomorphometry parameters as compared to ovariectomized group [1]. In this article, we reported the remaining half of the findings i.e. the dynamic and cellular bone histomorphometry parameters.

No significant differences were observed in single-labeled surface between the treatment groups. High single-labeled surface reflects high bone resorption rate [26]. Thus, the absence of significant differences in this parameter indicates that Ca 1% and *C. caudatus* did not affect the resorption process. However, this study showed that groups treated with Ca 1% and *C. caudatus* had higher double-labeled surface. The presence of high double-labeled surface indicates high bone formation and active mineralization process [27,28]. The results suggested that 500 mg/kg *C. caudatus* extract may play a role in stimulating bone formation in ovariectomized rats. Calcium, on the other hand, has been used as a treatment for osteoporosis [29]. Daily calcium supplementation has been shown to prevent bone loss in postmenopausal women [30].

Mineral appositional rate (MAR) is the distance between two fluorescence labels which were formed when calcein was injected on the ninth day and second day before rats were sacrificed [31]. MAR represents the osteoblastic activities in the bone [32]. High MAR value in the Ca and CC groups is suggestive of high osteoblastic activity. This is in agreement with osteoblast surface value which was higher in the Ca and CC groups compared to OVX.

Osteoid volume (OV/BV) was found to increase significantly in the sham and CC groups compared to the OVX group. The CC group also had a higher osteoid volume compared to the Ca group. Osteoid is bone matrix that is not mineralized yet. The increase in osteoid volume may occur due to the increase in osteoid formation or by the disruption that has occurred during the process of mineralization of bone [33]. The increase in osteoid volume in the CC group showed that *C. caudatus* may have a role in osteoid formation. This effect of *C. caudatus* is an advantage over Ca 1%. *Cosmos caudatus* was found to have high phenolic content of the flavonoids class (which include catechin, epicatechin, myricetin and luteolin) and was observed to possess free-radicals scavenging activity [34]. These properties of *C. caudatus* may also contribute to its bone-protective effects. It is also reported that *C. caudatus* contains 270 mg calcium per 100 g of the plant [35]. This composition, in addition to its antioxidative property, may contribute to the superiority of *C. caudatus* as opposed to calcium supplementation.

Calcium supplementation in this study was given in drinking water at the concentration of 1%. This method of administration was used in our previous study [36] and has also been used by others [37]. The advantage of using this route of administration is that it minimizes the stress from using injection or oral gavage. However we did not measure serum levels of calcium to ensure adequate calcium intake which can further verify the differences seen between the *C. caudatus* group and the Ca 1% group.

No significant differences in osteoid surface (OS/BS) were observed between all the groups. Although the percentage of osteoid surface is higher in the Ca and CC groups than in the OVX group, significant difference was not observed. This may be attributed to the small sample size used in this study.

There were no significant differences in osteoclast surface (Oc.S/BS) between all the groups. However, based on a previous study, there was a significant increase in Oc.S/BS for ovariectomized rats [38]. In addition, post-menopausal condition can stimulate the formation of osteoclast by the production of nuclear factor-kβ ligand (RANKL) and tumor necrosis factor (TNF) by monocytes and T cell, and this phenomenon tends to occur in women with osteoporosis [39]. *C. caudatus* did not seem to cause any effects on osteoclast surface which implies *C. caudatus* has no effects on resorption process.

## Conclusions

*C. caudatus* has beneficial effects on dynamic and celluar bone histomorphometry parameters in ovariectomized rats. *C. caudatus* was also observed to be superior to Ca 1% in increasing the osteoid volume parameter. Thus, *C. caudatus* has the potential to become an alternative treatment in restoring bone damage in post-menopausal women.

## Competing interests

The authors declare that they have no competing interests.

## Authors’ contributions

NM participated in the design of the study and drafted the manuscript. ZS carried out the analysis and performed the statistical analysis. ESMR participated in interpreting the bone histomorphometry analysis. NM helped in the design of the study and helped to draft the manuscript. All authors read and approved the final manuscript.
